# History of Diphtheria in the Town of Hamburgh and Vicinity, Erie County, N. Y., with Some Reflections Relative to Its Character, Treatment, Etc.

**Published:** 1867-02

**Authors:** George Abbott


BUFFALO

### Medical and Surgical Journal.

VOL. VI. FEBRUARY, 1867. No. 7.

### Original Communications.

ART. I—*History of Diphtheria in the town of Hamburgh and vicinity, Erie county, N. Y., with some reflections relative to its character, treatment, etc. Read before the Erie County Medical Society, January* 8, 1867, *by the retiring President,* GEORGE ABBOTT, M. D.

[PUBLISHED BY VOTE OF THE SOCIETY.]

In an address like the present, we have thought it not advisable to attempt to show the when, where, and how diphtheria came first to attract the attention of observing physicians, or the success attending the varieties of treatment adopted for its relief, as that and much more is equally within the reach of every member of the Society. Nor is it advisable we should attempt a rhetorical display, for before me are many who could eclipse by far any production I might have the honor to present. We hope, however, to interest you by a brief recital of facts and incidents connected with this disease, arising in my own practice and immediate observation. In medicine facts are everything, theory nothing, only so far as theory is deduced from fact, and is in strict accordance therewith. We will describe the symptoms, etc., of only some of the typical cases, denominating them as case 1st, 2d, and so on, and for the sake of brevity will refer the others to these.

In the month of September, 1859, upon the heights of those beautiful hills, lying some twelve or fifteen miles to the south-east of this city, called "chestnut ridge," and some five miles east of the village of White's Corners, [Hamburgh] two young ladies, the Misses Lockwood, suddenly sickened and died with what was called putrid sore throat. Several others in the neighborhood sickened in a similar manner, but recovered. I am told by one of the attending physicians that these cases were in all respects identical with what we have since called diphtheria.

On the northern slope of this same ridge, near its foot, some three or more miles from Lockwood's, and about the same distance from White's Corners, is the Littlefield neighborhood, in which, the last of this same month, a sore throat endemic broke out, attacking first on the 17th September Ella C., aged 6 years; the 22d, Pruda C. an adult; the 23d, Melissa, and the 24th, Mary C., sisters, aged 14 and 17; the 26th, Almon C., an adult; the 15th November John S., aged 14, and the 23d, his sister, some 8 years old.

Each of these cases were characterized by deep red, swollen tonsils and throat, with enlarged lymphatic glands, voice more or less affected; ash colored and ulcerated spots on tonsils, from which membrane was removed; in one case a piece described in my minutes as of the thickness of a case-knife blade, 1½ inches long by ⅝ wide, which came near suffocating the patient at the time of expulsion. As a sequelæ in two cases, there was some difficulty of vision, and in a third, for some months the swallowing of liquids would be followed with their frequent return through the nose. These cases occurred within the area of ½ or ¾ mile, and having attended them all, I know them to have been veritable diphtheria.

It is said in the spring following a fatal case occurred near Boston Corners, in the family of Orrin Lockwood; with that exception, our entire section of country enjoyed a complete immunity from this terrible scourge until the spring of 1861, when it reappeared in the Littlefield neighborhood, slightly at White's Corners, and 2½ miles below in the Grossman neighborhood.

Case 1st.—March 2d, 1861, was called to see Helen L., aged 2½ years; had been slightly sick a day or two, is very palid and feeble, some pain through sides of neck, head and chest, enlargement of the lymphatic glands under the ears, at the angle of the jaws; neck moderately stiffened, slight soreness of throat, and tonsils swollen, which, with the fauces are redder than natural. On the inner posterior surface of each tonsil is a patch of whitish ash colored membrane the size of a dime; breath fetid, no appetite, and is losing flesh and strength daily.* She had just returned with her parents from Dunkirk, where diphtheria was prevailing. The treatment consisted constitutionally, of quinine, iron and stimulants. Topically, swabbing the throat frequently with an astringent wash made of alum, borax and honey, and cauterizing the throat twice daily with a strong solution of nitrate of silver, also salt pork bound on the throat externally; diet, milk and soups.

Our little patient becoming especially worse at the end of the third or fourth day, we rejected the solution of nitrate of silver, substituting a very careful but thorough touching of the membranous spots with solid caustics. Improvement soon commenced, and she was discharged convalescent the 16th day. Three weeks after, she was taken with partial strabismus, severe neuralgic pains through her back and limbs, and considerable paralysis of the lower extremities, all of which soon yielded to a liberal use of quinine and stimulants. No other case occurred in this family or neighborhood until the following September.

The 24th of March Drs. Smith and Nott met the disease in the family of Frederick Graham. Amelia G., aged 4, was puny but playful the 22d and 23d, was taken severely ill the 24th, and died the evening of the 28th. Her brother Lewis, aged ten months, sickened the 30th, and died on the sixth day. Julia, aged 8, and Matilda, aged 6½, complained slightly the 30th, and were taken severely ill the first of April. Matilda died on the 12th, Julia, after three weeks' very dangerous illness, recovered. The father and mother were taken sick about the 11th or 12th April, convalesced in about ten days. The morning of the 28th a sister, Emma, aged 2 years, was taken to her uncle Harmon Graham's, more than a mile and a half north, on "Cooper ridge." She was taken the first of April and died the 14th. Harmon's son, aged 2 years, was taken the 10th, was dangerously sick fourteen or fifteen days and recovered. Harmon and his wife were also taken soon after, and convalesced about the tenth day.

* The expectoration of a slimy, tenacious saliva and epistaxis, though not mentioned in this case, are frequent attendants. The latter, especially, mark severe cases. A darkish, black or black-mottled appearance of the membrane is often seen in the severer cases and augurs badly.

On the 14th Theodore, aged 20, Caroline aged 16, and Hannah, aged 13, brother and sisters of Frederick and Harmon, were taken; the first two convalesced about the eighth day. Hannah was more dangerously sick, and did not convalesce under twenty days or more. There were fifteen members in the two families, thirteen of whom were sick as above stated, four dying. The exempted ones were two grandmothers, aged about 55.

I was called to this family on the 15th, and found the living and one dead, [Emma] at Harmon's house; nine then sick, similar to case 1st, four very dangerously. From that time they were treated with full doses of quinia, carbonate or tr. muriate of iron and chlorate of potash; topically, gargles of alum, sulphate of zinc, chlorate of potash and tr. mur. iron, with a thorough but very careful application of the solid caustic to the affected points, with the exception of Hannah, whose opposition to local treatment was so great that it was neither thorough nor persisted in. Her case assumed many of the severer symptoms soon to be described in case 3d. Her nervous system was much debilitated and presbyopia followed as a sequela. Harmon, Theodore and Harmon's son were quite afflicted with debility and derangement of the nervous system as sequela, but recovered promptly under the use of quinia.

On the 16th and 17th I took charge of the families of John Spitler, Jacob Zimmerman, Jacob Dryer and others, near neighbors of F. Graham, in all eight children, each attacked similar to case 1st; adopted the same described treatment, and all promptly recovered. It is a noticeable fact, that no communication was had between the house of H. Graham and his neighbors, and no other cases occurred in that neighborhood.

Case 2d.—April 13th, visited Mrs. Dingey, aged about 50, recently from a vicinity in Canada where diphtheria prevailed. Was taken like case 1st, except less enlargement of the tonsils and lymphatic glands in the neck. There was marked languor and debility of the nervous system, requiring the prompt administration of quinine and stimulants. Whenever the strength of the system was permitted to flag, a thin, delicate, gause-like membrane would spread from the throat over the roof of the mouth. The mucous surfaces underneath this membrane have a slight redness. To these shadows, if I may so term this delicate membrane, solid caustic was inapplicable; astringent washes would easily accomplish their removal. Within three days, four others in the same house were taken like case 1st. Under similar treatment they recovered in ten days.

By the first of May the epidemic had entirely disappeared, and with the exception of one or two slight cases we enjoyed an entire immunity from this disease until the following September, when it again appeared in the Littlefield neighborhood, at White's Corners and at North Boston.

On the 4th of September Dwight L., aged 6, and on the 19th his mother, were taken similar to case 1st. Both readily submitted to prompt and thorough topical treatment with solid caustics.—Dwight took moderate doses of quinia and tr. mur. iron, and convalesced in some two weeks. The nervous debility of the mother was much like that of ease 2d, and required full doses of quinia, iron and stimulants to bring her up,

Case 3d.—Alice L., aged 8, sister of Dwight's, was taken on the 7th of September. For the first five days her case did not appear as grave as his; indeed it was a mild form of case 1st, but she persistently and successfully resisted all applications to the tonsils and pharynx, not even gargling. She took two full doses of quinia, tr. mur. iron and chlorate of potash, still the disease steadily progressed, covering the tonsils and filling the throat and nostrils with membrane. By the 9th day the tumefaction of the sides of the neck was immense and extended nearly together in front, and across the back. The capillary circulation failing rapidly, purple specks and spots appeared and disappeared over the limbs and body.—Pulse irregular, at one hour perhaps 95, and another 120; very restless and wandering at times; breath exceedingly fetid and loathsome, respiration irregular and difficult on account of the filling up of the throat by the swollen tonsils and membrane, and death came to her relief the eleventh day. In this case the constitutional treatment was thorough, while the topical was a complete nullity.

Mrs. Sophia A., aged 37, naturally robust, lives three-fourths of a mile west, visited Alice the 17th. On the 19th taken like case 1st. Treatment: swab and gargle with preparations of alum, tr. mur. iron, and sulph. zinc. For nine days cauterized tonsils and pharynx, once daily. On account of a strong prejudice against it took only small doses of quinia for six or seven days, and she remained thin and pale and debilitated for several weeks. A free exhibition of quinia would have restored her usual health promptly.—The 28th October her daughter, aged 13, and the 12th November a second daughter, aged 16 months, sickened with the same form, mildly. Treatment: caustics and gargles only. Convalesced in five days.

October 1st, Roena C., aged 10, taken like case 1st, save it was ushered in with an unusual amount of high fever, chills and headache; was seriously ill. In less than twenty minutes after thoroughly cauterizing the spots, the apparently high fever passed off, and she remained cool and pale. Repeated the cauterization for six days, once or twice daily, as the case required. Afterwards less frequently; gave free doses of quinia, and she convalesced the sixteenth day.

November 14th, Mrs. L. B. Littlefield, relative of D. S. L., living eighty rods west. Her husband had visited the sick, but she had not. Is a slender, nervous woman, taken as case 1st, modified with less tonsilar and glandular enlargement, which is a common fact in adult cases. Treatment: cauterized twice daily, quinine and stimulants. Like case 2d, if the quinia was omitted the symptoms returned. Convalesced in eleven or twelve days. The 26th, Miss Lucinda L., an adult, taken and treated like case 2d, convalesced in twelve days.

Case 4th.—The 29th, Julia R. L., daughter of Mrs. L. B. L.. aged 5, attacked with cutaneous diphtheria. First appeared on the right cheek, as small, inflamed, blistered spots, which, when broken, discharged a yellowish, irritating water, the abraded or blistered surface covered with a whitish membrane. The whole cheek and skin red, swollen and inflamed. The glands of the neck enlarged, and all the common symptoms of diphtheria present, save internal membrane. Treatment, 30 drops of tr. mur. iron, daily, and an occasional dose of quinia. Topically we applied the solid nitrate of silver to the membranous spots, the effect of which would be to immediately dry it up, and abate the swelling and inflammation of the base. Occasionally it would re-inflame under the crust and crack open, requiring a repetition of the treatment. We experimented somewhat on some of the spots by using dry and moist tannin, alum and other astringents, but found nothing that would compare with the solid caustic in power to control the swelling and inflammation and spread of the blisters. She convalesced in two weeks.

December 15th, Mrs. Nancy L., aged 69, was attacked similar to case 1st, considerably modified. She had been tapped by Dr. G. N. Burwell of this city, for ascites, forty-six times within twelve years. Has had a sore throat several times of late, and to-day has also headache, feverishness and membrane on the tonsils. Used caustics; gave quinia liberally, and she soon convalesced. Two children in the family, to whom I administered tr. mur. iron, and an occasional dose of quinia, as a prophylactic, escaped any attack.

Case 5th.—December 10th, Mrs. John K., aged 30, living a mile east, was taken suddenly and severely, as follows: high fever, acute pain throughout the system, throat very red, deglutition difficult, quite a nasal twang to the voice, and most of the symptoms belonging to case 1st. The right pillar and anterior of the pallate resembles a great, well-filled leach, behind this, is a large strip of membrane running up and into the posterior nares. Treatment: caustics, twice daily; gargles, tr. mur. iron, and full doses of quinia. Convalesced in ten days. The 17th, her husband, who acted as her nurse, was similarly taken; took a thorough sweat, applied counter-irritants to the throat externally, gargled with iron, etc.; took a few doses of quinia, and he convalesced soon. A son, 3 years old, to whom we gave 25 drops tr. mur. iron, daily, and kept from the sick room, though with his mother the first 24 hours of her sickness, did not contract the disease.

The 17th of December Mrs. James A., a woman of frail constitution, was taken similar to case 2d, only more severely. Her son, a lad of 12, had frequently visited his sick aunt and cousins, Mrs. E. A. and daughters, living across the road; and two weeks ago had a bad sore throat, which they treated topically and was soon well. Her case is only remarkable for being complicated the tenth day with the appearance of the menses, which caused a grave reaccession of the symptoms, demanding an unusual amount of quinia and stimulants. Convalesced about the eighteenth day.

While cauterizing the patches I accidentally touched some healthy parts, and at the next visit found them covered with dense membrane. I have observed like results in other and similar instances.

The 30th and 31st, Abram C. and wife, aged 24 and 19, were taken like case 5th; reside one mile north. Mrs. C. was very sick, pulse ranging from 130 to 145 for ten days. She had a young child, which was permitted to nurse her some, but did not contract the disease. Perhaps we should mention with these, the cases of Mrs. T. C. A. and family, a sister of L. B. L.'s, though living some six miles distant, and the family of a sister-in-law, Mrs. L. H., three miles distant. No cases occurred in their neighborhoods outside of their families.

North Boston is a thickly settled school district in the valley of the Eighteen Mile Creek, near the termination of the east and west Boston hills, four miles above White's Corners, and has been rendered illustrious in the annals of medicine, by the occurrence of a remarkable epidemic of typhoid fever, some years since described and reported in the *Buffalo Medical Journal*, by Prof. Austin Flint.

The present epidemic was confined exclusively to the valley, a mile or so up and down the creek. I date its commencement with the sickness and death of Mary Fix, a German girl of ten summers, who, with several others, had her ears pierced about the 26th of September. In her case alone an erysipelatous inflammation followed, extending rapidly from the left ear upon the side of the neck and front of the chest. I found her on the 29th with a highly fevered cheek, very white lines about the nose and mouth, furred tongue, pulse 140; though she rose from her bed and had a bright eye. On the parts described was an apparently blistered surface, averaging six or seven inches long, by two or three wide, covered with a dense white membrane, upon a wide spreading, angry, inflamed base. They assured me it was wholly the effect of disease. We diagnosed a traumatic erysipelas of malignant type, and prognosed speedy death, unless our efforts were rigorously carried out and remarkably successful. Suffice it to say, another physician, summoned previous to, but coming after me, prognosed favorable results, and no danger. My treatment was suspended, and she died in thirty hours. I surmised that this might be what Bretonneau called "Cutaneous Diphtheria," but never having found any description of that form of the disease, nor seen anything of like character before, I was unwilling to declare such a diagnosis. Experience and experiments have since confirmed me in the belief that it was a case of genuine cutaneous diphtheria.

The day following Mary's death, her mother sickened with mild diphtheria; convalesced in six days. The 15th October her sister 18 months old, was taken like case 1st. Adopted the topical and tonic treatment, and she convalesced in ten or twelve days.

I was told that Mary L., aged 13, and Miss Calista S., aged 21, visited Mary F. before and after death, both sickened within three days with diphtheria, and died in about a week.

The 11th October I was called to assist Dr. Davis, an excellent physician of the town of Boston, in the care of Miss Cynthia L., aged 16, sister and nurse of Mary F. She sickened the 8th, similar to case 5th, complicated after the fifth day, with cutaneous diphtheria. The Doctor had adopted what is usually called a vigorous topical treatment, to wit: cauterizing the throat with a solution of nitrate argenti, about 30 grains to the oz., and constitutionally, had exhibited tr. mur. iron, chlorate of potash, Dover's powder, etc.; still the disease was gaining ground rapidly. We amended by substituting the solid for the dissolved caustic, and adding quinine and stimulants. The solid caustic was applied thoroughly to the cutaneous difficulty, leaving, however, daily for experiment, a point of the blister not larger than a pin's head untouched. From this untouched point the inflammation and blister would spread rapidly, while the remainder dried immediately up and healed kindly. She convalesced the fourteenth day.

By the Doctor's courtesy I visited Miss S., mentioned previously. The attack was similar to case 1st, and progressed to a rapid fatality. Also visited two of F. Neste's daughters, in one of whom the right meatus auditorius externus was lined with membrane; both convalesced. The mother and the girls were often in to see Mary Fix. On the 18th and 19th I took charge of three other young daughters of Mr. Nestes, attacked like case 1st, complicated in one patient, with a very severe cutaneous diphtheria, contracted in a small wound in the palm of the hand. It was successfully controlled with solid caustic, my usual treatment. All convalesced about the twelfth day.

The 15th, Mrs. C. and eldest girl attacked mildly, and recovered with little treatment. The 16th, L. W., aged 3, and the 19th, Miss Eunice C., aged 18, very severely. These were similar to case 1st. Same treatment, and recovered in twelve or fifteen days. Mr. Fix's residence was a hotel, post office, grocery, and place of general resort for all the neighborhood. The above cases were his immediate neighbors. About the same time it broke out among his more distant neighbors, and within a short time was terribly prevalent throughout the whole district. It continued to rage for over three months, attacking 56 of its 100 children, bearing 13 of them to the grave. Most of the nurses in attendance on the sick had mild attacks, though only one adult, Miss S., died.

The 15th of September it appeared at White's Corners, in the person of Emegene F., aged 5. Her attending physician, depairing of her life, the 23d, I was solicited to take charge of her case. Found her pale and bloodless as a corpse; pulse 120, very irregular and intermittent; much paralysis of the palate, nearly destroying her speech, and seriously impeding her respiration and deglutition. The mouth and nose were lined with membrane, and all the symptoms were present which are met in a slightly modified form of case 3d. We injected the nose and fauces with strong astringents, tr. mur. iron, smarting her throat severely. We gave her but little, chlorate of potash promoting diarrhœa, it was suspended. Quinine was taken and borne in large doses. To see the steady and progressive regulation of the pulse, as the system came under the influence of that powerful tonic was wonderful. Still more instructive and interesting was it to notice the quick return of its irregularity and intermission whenever the dose was diminished. By astringent injections we endeavored to keep the membrane well condensed, and it soon began to be dislodged. Her slow convalescence was attended with an extension of the paralytic symptoms to the entire system, from which she in time recovered. We attribute her salvation to the judicious administration of that inestimable tonic, quinia. The remainder of the treatment was too trifling to be aught but mild auxiliaries.

The 17th, Flora and Mary L., aged 8 and 6, similar to case 1st. Treatment: caustics, quinia and iron gargles. Convalesced in eight or ten days. October 10th, George B., aged 3. Treatment:

quinia, tr. mur. iron, and chlorate of potash, The topical treatment was very hesitating and inefficient—almost a nullity. The disease progressed similar to case 3d, and he died the eighth day. Also, same day, three young children of Mr. C.'s. November 14th, Josephine S., aged 12, and George L., aged 3. These last similar to case 1st. Cauterized as each required. Administered gargles and quinia, and cenvalescence followed promptly. There were a few other cases in the village, and I am told a couple of deaths. The last of October it is said to have made its appearance in a German family at Big-tree Corners, four miles north of White's Corners.

Case 6th.—At this time, Mary C., aged 16, a near neighbor, was lying very ill with typhoid fever of fourteen days. Dr. G. F. Pratt, one of the oldest and most eminent physicians of Western New York, saw her in consultation the 1st of November, pronounced it an uncomplicated case of typhoid fever, with an excellent prospect of recovery. At 5 o'clock next morning the attendants notice some hesitancy in her swallowing. At 11 A. M., for the first, a thin, whitish membrane was found all over the mouth. At 12.30, it was thick and dense, and easily removed with astringent washes. At 3 P. M. was seen by Dr- Pratt, (his second consultation,) and he called it pure diphtheritic membrane. She sank rapidly in spite of the most strenuous efforts, and died at 11 P- M., just eighteen hours after the first intimation of a diphtherial complication.

The 3d of December it again appeared on the creek, three-fourths of a mile above Graham's.

Case 7th.—December 11th, 11 P. M. was hastily called to see Adam F., aged 7. Has complained of sore throat for eight days and breathed croupy for three nights. Since 4 P. M. has grown "hoarse and hard to breathe;" breath fetid, sides of neck moderately swollen, tonsils enlarged and covered with membrane; also is a strip of the same one-half inch wide in the posterior fauces, extending upwards and downwards beyond vision; voice a whisper; cough croupy; pulse 122. Treatment: cauterized the tonsils and pharynx; swab the throat every three hours with alum, vinegar and honey; gargle with alum and iron water alternately; quinia and chlorate of potash full doses, croton oil and turpentine externally to the neck.

December 12th, 9th day. Rested and breathed better last night than any night previous. Has raised many pieces of membrane; one shown me was one-third by three-fourths of an inch long and thick as a case-knife blade; tonsils and fauces much cleaner and breath sweeter, but voice suppressed; added to the treatment inhalation of steam of salt and vinegar.

December 13th.—Breathes with the utmost difficulty, reaching about and seizing the bedding for help; is extremely blue. An effort to swab the larynx with a strong solution of nitrate of silver relieves the breathing considerably. 7 P. M.; has rested well through the day; had one paroxysm of strangling severely, which was relieved by expectoration of membrane; cough looser and voice more audible than before swabbing the larynx.

Dec. 14th, 3 A. M., 11th day.—Sweats profusely, labors hard at each inspiration and expiration; circulation is very feeble; cannot live long thus. 6 A. M. The brave little fellow, unassisted, stood between his father's knees, perfectly motionless, while I opened the trachea. He breathed easily, was immediately comfortable, and remained so throughout the day. The trachea was found lined with membrane full one-half line in thickness. 7 P. M. Has frequently expectorated portions of membrane, requiring prompt assistance to prevent strangulation. As I was cleaning the orifice a cough was excited, which expelled several pieces, one of which caught in the passage; he strangled, and before its removal could be effected, was dead. It proved to be near the half size of a chestnut. His sister, aged 18, who nursed him for three days, was taken severely, and his mother and some of the children, mildly, the 15th; similar to case 1st. Treated them with caustics, gargles and quinia; all convalesced in from four to nine days.

During the sickness Mrs. F. called at a neighbor's, and some five days after they commenced to sicken, one after the other, until seven had been attacked, all similar to case 1st. Same treatment and favorable termination.

For two or three years following, frequent sporadic cases occurred in different parts of the town, almost wholly confined to the changeable portion of the year. A history of its development and spread in some families would be especially interesting, but time will not permit. As an epidemic it may be said to have disappeared with the winters of 1561, '62 and '63.

We attempted to tabulate some 200 cases, but time has not allowed the completion of only the first 160. These show that September has furnished me 13, October 29, November 14, December 30, January 4, February 1, March 3, April 23, and June 2 cases.

It is especially noticeable that the most changeable months of the year are the most prolific of this disease. Damp weather, accompanied or followed with bleak, raw air, eminently assists its sporadic development and easy propagation.

The cases already presented, (and we have many others equally as pointed,) certainly warrant us in believing it contagious; not to the extent or degree of variola or rubeola, but more like that of scarlatina, with which indeed it is so often connected, that I have frequently called them twin-sisters. They impart to each other a degree of malignancy sometimes truly dreadful.

I might cite many instances where scarlatinous diphtheria, or diphtheria with a rash, desquamation and albuminuria, has developed ordinary diphtheria, and vice versa, but will mention only one. Hiram W-, near Springville, had a small daughter, who arose apparently well; before she had swallowed anything commenced vomiting violently; soon broke out all over with a scarlatinous rash; glands of neck swelled rapidly, throat filled with membrane of the ulcerated or sloughy character, and when I first saw her, at 3 P. M., was unconscious and moribund. Died at 7 P. M., twelve hours from apparent health. Four days after a second daughter was similarly taken, living only sixteen hours. A son and elder daughter were taken same day, and the mother the day after, with genuine or uncomplicated diphtheria.

The disease being contagious, it must have a period of incubation. In 59 cases we have been able to approximate closely the day of exposure. In them we find the extremes to be 2 and 14 days; the mean, $5\frac{2}{3}$. Of those under 20, the mean is $4\frac{3}{4}$; over 20, $7\frac{1}{3}$ days. In one instance a daughter sickened 34 days after the mother's convalescence, and I have denominated it a sporadic case. I am confident this period may be lengthened and the attack kept at bay, or often aborted by the use of tr. mur. iron and quinia, with proper protection against the changes of the weather.

Of those attacked 2 years old and under, 8 were males and 9 females; from 3 to 10, 25 males and 50 females; from 11 to 20, 9 males and 25 females; over 20, 11 males and 26 females. Total, 53 males and 110 females. The male cases ending in recovery, had an average continuance of $7\frac{1}{2}$ days, ending in death 6 days, while the female cases recovering averaged 10, and those dying $9\frac{1}{2}$ days. Of the fatal cases, 5 males and 4 females, 3 were like case 3d, received constitutional but no topical treatment. In three others the topical treatment, to wit: swabbing, gargling, etc., to be attended to by the nurses, was extremely inefficient, and the morale of the patient thereby so reduced that I could make but slight topical applications, and that too only occasionally, and would have abandoned them but for the urgent solicitation of some of their friends. Of the remaining three, one—case 7—died by accident. A second, when first seen, had been croupy for several days, and the mouth was filled one-third full with the swollen tonsils and membrane. Forty hours from my first visit he was in articulo mortis. I was entreated to try the experiment of opening the trachea, and did so. The little fellow was immediately restored to consciousness, and lived in comparative comfort for twelve or fifteen hours. The third contracted the disease from his sister, who died twelve hours from her attack, with scarlatina or scarlatinous diphtheria. The treatment exercised a marked mitigation of symptoms, though he died the fifth day.

Case 6th, is introduced and described as essential in showing the manner in which this disease will sometimes engraft itself upon others; but being of no value in the table of diphtheritic mortality, it is not placed among them.

Above the age of three, the liability of females as compared with males to contract the disease, is as two to one. Females being so much more exposed to the contagion in their attendance on the sick, will probably account for their greater ratio of attack. The chances of recovery are slightly in favor of the males.—Eleven are reported to have a second attack—two within three weeks and one four years after. Eleven are also reported as requiring medical assistance for paralytic symptoms, varying from moderate nervous debility to almost idiotic imbecility.

I never saw a case presenting the persistent symptoms of diphtheria, but membrane would be found upon a thorough examination; therefore we have recognized none as diphtheria, without the presence of membrane and an affection of the glandular system. The membrane is of a porus texture and absorbs the effusion of the part where located, and being within, still measurably independent of the animal economy, it speedily undergoes putrefaction, and causes an intense fetidity of the breath, and rapid poisoning of the blood. I have met with it from the thickness of the thinest gauze, like that now shown you, from the buccal mucous membrane of Miss L. L., in 1861, to that of "chunks," large and thick as the half of my thumb, like this, expelled from the throat of Miss E. F. in 1861. The alcohol in which it is preserved has condensed it to less than one-eighth its original size. Caustics and astringents operate upon it in like manner, and so harden it that it can usually be easily removed, or by its condensation putrefaction is checked or prevented, and the breath loses its fetidity.

There are other reasons why this membrane should be removed. If allowed to remain it soon attaches itself to the mucous tissue, first by points, finally throughout its under surface, and then any breaking or starting of it up or off, may be attended with a constant oozing and escaping of blood in the discharges from the nose and mouth, or again through the abraded surface underneath the membrane a constant absorption of the poison generated in it takes place. Thus we see that the presence of membrane is a prolific source of blood poisoning, constantly making its entrance through the respiratory and capillary systems. Shall we wonder at the enormous anlargement of the lymphatic glands, or the sometimes sudden failure of the nervous powers?

I am well aware that a strong prejudice exists with many in the profession against topical treatment. My experience forces me to believe it is the Herculean club by which we can meet and most effectually despoil this hydra-headed monster. Allow me to suggest, as a query, whether this prejudice may not be based upon returns made by insufficient experience or inefficient practitions, who, using once or twice daily, a solution of nitrate of silver, or perhaps other moderate remedies, with no benefit, as in the case of Helen L., Miss S. L. and Miss C. S., or using the solid caustic, thrust it into the throat and poke it about, like a blind man in the dark, as I have heard patients tell of Doctor's doing, thus injuring sound tissue, and finding the disease accelerated rather than retarded, reports topical treatment a failure, and yet inadvertently acknowledge its ability by exhibiting washes, chlorate of potash, and divers other gargles.

We have seen that the presence of membrane is a source of rapid blood poisoning. Therefore why not condense or remove it? One objects, you will irritate the parts; another, that it will return again; a third, that to the patient it is an unpleasant and usually an unwelcome procedure, and if not attended with a prompt recovery, is likely to bring discredit upon the practitioner.

To the first I answer: often the operation affords relief, but should the practitioner roughly use his probang, loaded with improperly prepared, or irritative medication, he would be more likely to do injury than good. Hence much depends upon the knowledge and skill of the physician. Again, I admit it will often return and repeat its return, usually, however, to a less and less extent. Perhaps it will increase in spite of your endeavors, but you will have the proud satisfaction of finding you have materially sweetened the breath and sensibly retarded the progress of the disease.

The third is hardly worthy of a reply, but I must confess I fear it is the most potent reason that influences objections. The want of "back-bone" to enable one, in the face of opposition, to stand up and do the thing which science dictates; a fearfulness of resbonsibility, a desire to screen one's self behind the whims and prejudices of the disordered intellect of the patient, and the natural but ignorant sympathy of the friends, leads him to pander to their notions and endeavor to secure by professional craft, what his knowledge, skill and integrity does not warrant him in possessing, to wit: the good will and confidence of his patron. We had designed making an analysis of treatment, illustrated by cases, for the purpose of showing the comparative benefit of the topical or non-topical course, but time will not permit. We will glance slightly at the remedies in use and close.*

---

* Observing that removal of the exudation, and the application of remedies to the subjacent surface, neither shortened the duration nor sensibly modified the progress of the complaint, but that the false membrane rarely failed to be renewed in a few hours, I very soon discontinued this rough local medication to the tender and

The previous history being that of our early experience, the solid caustic figures largely. In the hands of a careful and thorough practitioner we believe it an invaluable remedy, but unfortunately upon internal surfaces, it requires an amount of skill possessed by but few. Upon external or cutaneous diphtheria, we have shown in the cases of Miss S. L., J. R. L., and F. N-, that it possesses a safe, positive and controlling influence. We might cite many others, I will however only relate my own. In the midst of an active epidemic I received a slight pin-scratch in the palm of my hand, ordinarily of no consequence. The second or third day it began to inflame and blister, the redness extending up the wrist and arm. Membrane formed within the blister, and the whole became quite painful; milder means on a two days' trial doing no good, I thrust into and about the blister the solid caustic. After two or three applications it healed kindly.

With children and timid persons its use is attended with so much difficulty that I early sought and experimented to find some less objectionable remedy. Ordinary astringent washes, alum, tannin, and the like, do well in the removal of deposits like that in case 2d or 6th, save when the mucous membrane had become abraded or ulcerated. In the so-called solution persul. iron, which is a powerful astringent of such slight escharotic properties as to require but little skill in its use, was found an excellent substitute. But the great amount of acid entering into its composition rendered it often quite irritative, consequently objectionable. I therefore applied to Dr. E. R. Squibb of Brooklyn, probably the most eminent and reliable chemist in the United States, to produce for me a preparation of iron, containing, if possible, more tonic and

---

already enfeebled mucous membrane. The propriety of this course became evident at the very first *post mortem* examination I had the opportunity of witnessing, and has been confirmed by all my subsequent experience. (*Greenhow on Diphtheria, page* 155.)

Nitrate of silver may also be employed in the solid form, but this we should not advise, particularly in the case of children. Nitrate of silver in this form has the disadvantage of creating a more decided eschar than the solution, stimulating the diphtheritic exudation and thus hindering the perception of the progress of the disease. (*Slade on Diphtheria, page* 142.)

The unconditional and absolute recommendation of nitrate of silver as a local application, and the equal confidence in quinine as a constitutional remedy, appears remarkable; especially so at this late period in the history of this disease, when physicians throughout the country, are trusting to milder measures, and generally observe diphtheria progress favorably without much interference.—Ed.

astringent, and less acid properties than the mis-termed solution persul. ferri. In reply he said, "I have long looked for some physician who would test in practice the minus acid preparations of iron," and sent me an unofficinal article, which he proposed to call "Liquor Sub Sulphate Ferri Minus," a beautiful preparation of greater astringency, equal tonicity and far less acidity than the persulphate, and devoid of escharotic properties. Whenever used it has given me the best of satisfaction. I believe it safe in use and worthy of trial whenever astringency and tonicity are required.

The amount of iron may be varied, as the case demands, and the preperation reduced with water to any consistency required. The best swab is a wisp or pledget of cotton wool, in a steel probang, or a piece of old, long, soft, raveled cotton or linen, tied on a stick. Eschew sponges and sponge probangs; they are altogether too harsh. Under this course, topical treatment is stripped of nine-tenths of its bug-bear severity, and the patient no longer bears to it his former repugnance. Though many cases may and do recover under the use of topical remedies alone, the nervous system is so often and rapidly effected that in all severe cases we must resort to its prompt support, with tonics, stimulants and concentrated nourishment. Of all the host of tonics, iron unexcepted, I have found nothing equal to the sulphate of quinine for the relief of the nervous derangement or paralytic symptoms. I will cite, in illustration, but three of many cases; first, that of Emegene F., already related; second, T. G., naturally a robust, energetic man, two months after an attack like case 1st, applied for relief. Has for some time past been forced to take at every third to tenth or fifteenth respiration, a long, deep, involuntary breath; is not yet able to work. Took from 15 to 20 grains quinia daily, and in less than ten days was not only permanently relieved, but could endure more work than any of his brothers. J. H., aged 4, general paralysis following an attack like case 1st. Can neither walk, stand, hold up his head, or hardly sit down; looks and acts idiotic.—Administered quinia. With each dose could see a visible improvement. On the third day getting out of the remedy, the symptoms commenced returning; re-administered and continued the quinia for one week, and he was well.

My experience does not warrant me in recommending chlorate of potash, either as a gargle, for iron is better, or as a constitutional remedy, for it will promote a diarrhœa. I have never been satisfied with its therapeutic action in this disease. Tr. mur. iron, when well borne upon the stomach, has given me great satisfaction, especially as a prophylactic. I have frequently known it and quinia to apparently abort an impending attack. As an external application to the neck, croton oil and turpentine, salt pork, or a cloth dipped in brine and bound around it for the purpose of moderate counter-irritation is excellent; it should be well protected from all changes of air.

Without wishing to enter into the discussion of the humeral pathology, I will say: If we admit any force to the theory and experiments of Prof. Palli of Milan, ably seconded by those of Dr. De Ricci of Dublin, that zymotic diseases are developed by a species of blood fermentation, and that this fermentative process may be safely checked by the administration of the sulphites and hyposulphites of soda and magnesia, which are reputed to be perfectly harmless, even in immense doses, I would recommend their trial in this disease.

Gentlemen, for the many honors you have conferred upon me, since my connection with this Society, and for the patient attention you have given on this occasion, I can but express to you my sincere and heartfelt thanks.

